# Common and unique multimodal covarying patterns in autism spectrum disorder subtypes

**DOI:** 10.1186/s13229-020-00397-4

**Published:** 2020-11-18

**Authors:** Shile Qi, Robin Morris, Jessica A. Turner, Zening Fu, Rongtao Jiang, Thomas P. Deramus, Dongmei Zhi, Vince D. Calhoun, Jing Sui

**Affiliations:** 1 Tri-Institutional Center for Translational Research in Neuroimaging and Data Science (TReNDS), Georgia State University, Georgia Institute of Technology, Emory University, Atlanta, GA 30303 USA; 2 Department of Psychology and Neuroscience Institute, Georgia State University, Atlanta, GA 30302 USA; 3 Brainnetome Center and National Laboratory of Pattern Recognition, Institute of Automation, Chinese Academy of Sciences, Beijing, 100190 China; 4 University of Chinese Academy of Sciences, Beijing, 100190 China; 5 Institute of Automation, Chinese Academy of Sciences Center for Excellence in Brain Science, Beijing, 100190 China

**Keywords:** Heterogeneity, Autism spectrum disorder, Asperger’s disorder, Pervasive developmental disorder-not otherwise specified (PDD-NOS), Autistic disorder, Multimodal fusion

## Abstract

**Background:**

The heterogeneity inherent in autism spectrum disorder (ASD) presents a substantial challenge to diagnosis and precision treatment. Heterogeneity across biological etiologies, genetics, neural systems, neurocognitive attributes and clinical subtypes or phenotypes has been observed across individuals with ASD.

**Methods:**

In this study, we aim to investigate the heterogeneity in ASD from a multimodal brain imaging perspective. The Autism Diagnostic Observation Schedule (ADOS) was used as a reference to guide functional and structural MRI fusion. DSM-IV-TR diagnosed Asperger’s disorder (*n* = 79), pervasive developmental disorder-not otherwise specified [PDD-NOS] (*n* = 58) and Autistic disorder (*n* = 92) from ABIDE II were used as discovery cohort, and ABIDE I (*n* = 400) was used for replication.

**Results:**

Dorsolateral prefrontal cortex and superior/middle temporal cortex are the primary common functional–structural covarying cortical brain areas shared among Asperger’s, PDD-NOS and Autistic subgroups. Key differences among the three subtypes are negative functional features within subcortical brain areas, including negative putamen–parahippocampus fractional amplitude of low-frequency fluctuations (fALFF) unique to the Asperger’s subtype; negative fALFF in anterior cingulate cortex unique to PDD-NOS subtype; and negative thalamus–amygdala–caudate fALFF unique to the Autistic subtype. Furthermore, each subtype-specific brain pattern is correlated with different ADOS subdomains, with social interaction as the common subdomain. The identified subtype-specific patterns are only predictive for ASD symptoms manifested in the corresponding subtypes, but not the other subtypes.

**Conclusions:**

Although ASD has a common neural basis with core deficits linked to social interaction, each ASD subtype is strongly linked to unique brain systems and subdomain symptoms, which may help to better understand the underlying mechanisms of ASD heterogeneity from a multimodal neuroimaging perspective.

**Limitations:**

This study is male based, which cannot be generalized to the female or the general ASD population.

## Background

Autism spectrum disorder (ASD) represents a broad range of pervasive developmental disorders characterized by limitations in initiating and sustaining social communication and interactions, in addition to the presence of repetitive behaviors [1]. In the past 50 years, the diagnosis of autism has evolved from a narrowly defined and rare disorder of early childhood, to a broader spectrum disorder representing lifelong challenges–with > 1% of children diagnosed with the disorder, and highly heterogeneous nature, i.e., no two children or adults with ASD have the exact same phenotypic profile [2]. Multi-level heterogeneity [3] across biological and behavioral attributes including genetics [4, 5], neural systems [6, 7] and clinical phenotypes has been observed in individuals with ASD. Individuals with ASD differ across multiple dimensions in their clinical presentation, including: the severity of specific behavioral deficits such as in social skills, communication and language ability, age of onset, intellectual functioning [8], sex differences [9–12], executive functioning [13] and developmental histories [14, 15]. Thus, ASD is frequently conceptualized as an “array of spectra” rather than a single phenotype. Many clinicians and researchers still hypothesize that it is comprised of different subgroups or multidimensional nodes or subdivisions [7, 16, 17] that are thought to have potential etiological and treatment value, although these have yet to be clearly identified and operationalized. Such remarkable heterogeneity across multiple levels of dimensions reflects a core challenge underlying the neurobehavioral modeling of ASD, or its subtypes.

The heterogeneity inherent in ASD [18] presents a substantial challenge for prevention, diagnosis and treatment [19]. Several studies have suggested that a biomarker approach which stratifies or partitions the ASD population’s variability into neurobehaviorally meaningful subgroups may aid in improving the identification of etiologies, early identification, diagnosis and its precision treatment, in contrast to a “one-size-fits-all” approach [16, 19]. Neuroimaging studies have indicated lower gray matter volume (GMV) in prefrontal gyrus and limbic striatal as compared to healthy controls [20] in Asperger’s subtype. Instead of categorical subtypes, a recent study proposed a continuous interindividual variation in ASD yielded three factors with dissociable whole-brain hypo- and hyper-resting state functional connectivity in default mode network [17]. Another study classified ASD into three distinctive anatomical subtypes (ASD-I: cortical thickening, increased surface area, tissue blurring; ASD-II: cortical thinning, decreased distance; ASD-III: increased distance) based on cortex-wide MRI markers [7]. However, they are both focusing on defining new ASD subtypes by classifying neuroimaging features into different clusters [21]. Here, we provide a different view regarding ASD subtypes by changing from defining new subtypes to directly compare the exiting subtypes in ABIDE data based on DSM-IV diagnostic system to investigate whether there are the commonality and differences among three subtypes. Although many neuroimaging studies have investigated ASD heterogeneity across sex, age and intelligence levels [10, 16], few have investigated ASD heterogeneity from a multimodal brain imaging perspective [22, 23]. In this study, we hypothesize that the heterogeneity of behavioral symptom severity, assessed via the Autism Diagnostic Observation Schedule (ADOS) [24], will provide another window into the heterogeneous, abnormal multimodal covarying patterns in individuals with ASD, and potentially allow for a comparison of similarities and distinctions among its traditional subtypes.

Although the concept of subtyping in ASD has long been utilized and has generated considerable controversy [25, 26], the neurobiological basis and the mechanisms underlying brain heterogeneity of potential ASD subtypes remain poorly understood. It is not currently clear whether ASD has a common core neural basis, or whether the disorder differentially affects various brain systems leading to individual differences related to the unique amalgam of deficits in abnormal social behavior, impaired communication and stereotypical behaviors, or some combination of a common core along with differential neural subsystem deficits, which might be suggestive of neurobehavioral substrates/subtypes. In order to identify such stratification or dimensional biomarkers in ASD, we change the approach from the case–control model to a stratified and dimensional model which better model the multidimensional neural and related behavioral attributes that could yield much higher impact, larger effects and result in the identification of core and unique neural behavioral substrates [27].

In this study, multimodal [28] brain imaging data from the Autism Brain Imaging Data Exchange (ABIDE) [29, 30] were used as the discovery (ABIDE II) and replication (ABIDE I) cohorts. Cumulating evidence has demonstrated that ASD reflects fundamental differences in both brain structure and function [31–33], while fractional amplitude of low-frequency fluctuations (fALFF) and GMV are two representative measures of (functional magnetic resonance imaging) fMRI and (structural MRI) sMRI. FALFF can directly provide information of the amplitude of brain activity of each brain region within a network, i.e., reveals the BOLD signals change of the regional spontaneous activity [34], which is widely used for characterizing local spontaneous activity of resting state fMRI data. Studies show that ASD exhibited reduced fALFF in the right middle occipital gyrus, lingual gyrus and fusiform gyrus [33]. And further spatial overlap analyses confirmed that the spatial pattern of reduced fALFF substantially overlapped with that of local under-connectivity [33]. ADOS scores were used as a reference to guide a 2-way fALFF-GMV fusion [35] to identify multimodal brain networks that were associated with the overall ADOS scores. For this investigation, we focused on four goals: (1) to identify multimodal brain networks associated with overall ADOS scores within ASD and among its subtypes (Asperger’s, pervasive developmental disorder-not otherwise specified [PDD-NOS] and Autistic); (2) to assess group differences between ASD/subtypes and typically developing controls (TDC) of these identified networks; (3) to identify the common and unique multimodal neurobehavioral attributes identified among the Asperger’s, PDD-NOS and Autistic subgroups; and (4) to evaluate the predictability of the identified brain networks in predicting ADOS and Social Responsiveness Scale (SRS) total scores in an independent sample (ABIDE I).

## Methods and materials

### Participants

Subjects with ASD (*n* = 229) and the corresponding TDCs (*n* = 126) were selected from the ABIDE II [29, 30], which is hosted by the 1000 Functional Connectome Project/International Neuroimaging Data-sharing Initiative (INDI) from multiple international sites (9 sites, Additional file 1: Table S1). Inclusion as a participant with ASD required a clinician’s DSM-IV-TR diagnosis of Autistic disorder, Asperger’s disorder or PDD-NOS. Each site followed slightly different methods for diagnosing patients with autism or ascertaining typical development; however, most of the sites used the ADOS and Autism Diagnostic Interview-Revised (ADI-R) scores to diagnose ASD. Specific diagnostic criteria for each site can be found at https://fcon_1000.projects.nitrc.org/indi/abide/. ADOS score is considered the “gold standard” for assessing Autistic children [36, 37] and is a semi-structured interactive evaluation of ASD symptoms that is used to measure social and communication abilities by eliciting several opportunities (or “presses”) for observing spontaneous behaviors (e.g., eye contact) in standardized context. The SRS, another valid quantitative measure of Autistic traits, was designed primarily to assess social reciprocity deficits. The ADOS total symptom scores were summed over “Communication,” “Reciprocal Social Interaction” and “Stereotyped Behaviors and Restricted Interests” based on the scores of the algorithm items of ADOS-G, in ways representing a severity dimension of key behavioral attributes. TDC were selected from the same ASD acquisition sites. Within the current merged ASD sample, there were Asperger’s (*n* = 79), PDD-NOS (*n* = 58) and Autistic (*n* = 92) diagnosed subjects from the ABIDE II database. Selection criteria for these ABIDE II subjects also required them to be younger than 35 years of age and males only (due to the low number of female with ASD) [38]. Basic demographic, symptomatic scores and medication use of participants are summarized in Table 1.

**Table 1:** Demographic and clinical information of participants

	ASD	Asperger’s	PDD-NOS	Autistic	ANOVA
ABIDE II					
Sample size	*n* = 229	*n* = 79	*n* = 58	*n* = 92	Na
Age (mean $\pm$ std)	10.0 $\pm$ 4.2	16.3 $\pm$ 6.4	8.3 $\pm$ 2.5	12.2 $\pm$ 3.8	7.0e−19
Handedness (R/L/M)	178/18/33	67/7/5	36/4/18	75/7/10	0.04
Intelligence (mean $\pm$ std)	106.3 $\pm$ 18.1	110.4 $\pm$ 15.6	104.4 $\pm$ 17.5	103.8 $\pm$ 20.0	0.04
Mean FD (mean $\pm$ std)	0.4 $\pm$ 0.2	0.1 $\pm$ 0.2	0.1 $\pm$ 0.2	0.5 $\pm$ 0.1	0.4
ADOS (mean $\pm$ std)	12.3 $\pm$ 4.9	10.1 $\pm$ 4.0	12.6 $\pm$ 4.6	14.1 $\pm$ 5.0	7.2e−06
SRS (mean $\pm$ std)	86.8 $\pm$ 29.7	81.5 $\pm$ 26.5	88.1 $\pm$ 29.4	92.0 $\pm$ 32.9	0.1
Number on medication	35	0	0	35	Na
ABIDE I					
Sample size	*n* = 400	*n* = 73	*n* = 28	*n* = 299	Na
Age (mean $\pm$ std)	16.2 $\pm$ 6.2	16.1 $\pm$ 7.2	16.0 $\pm$ 6.7	16.2 $\pm$ 5.9	0.97
Handedness	230/28/144	47/4/22	16/4/8	167/20/112	0.36
Intelligence (mean $\pm$ std)	104.2 $\pm$ 16.7	112.6 $\pm$ 15.8	100.2 $\pm$ 23.1	102.5 $\pm$ 15.6	1.3e−05
Mean FD (mean $\pm$ std)	0.16 $\pm$ 0.2	0.2 $\pm$ 0.2	0.1 $\pm$ 0.07	0.2 $\pm$ 0.2	0.3
ADOS (mean $\pm$ std)	14.0 $\pm$ 4.7	12.2 $\pm$ 4.2	9.4 $\pm$ 3.3	14.5 $\pm$ 4.7	0.003
SRS (mean $\pm$ std)	90.6 $\pm$ 30.4	82.5 $\pm$ 20.5	90.1 $\pm$ 28.8	92.0 $\pm$ 31.9	0.4
Number on medication	83	0	0	83	Na

ANOVA column presents the *p* values for ANOVA test among Asperger’s, PDD-NOS and Autistic subgroups. For handedness (categorical measures), Chi-square was applied

*FD* framewise displacements, *Intelligence* full scale IQ

The same ASD selection criteria were used for inclusion in the cross-validation ABIDE I (17 sites, Additional file 1: Table S1) dataset, which included Asperger’s (*n* = 73), PDD-NOS (*n* = 28) and Autistic (*n* = 299) diagnosed subjects. ABIDE II was selected as the discovery cohort due to its comparable sample size among the three subgroups as well as the small site number (9 vs. 17), comparing ABIDE I (replication cohort). There is no significant correlation between the referenced ADOS scores with age, handedness, mean framewise displacements (FD) and SRS (Additional file 1: Table S2). The group differences between ASD and TDC, and between subtypes and TDC on key clinical measures are provided in Additional file 1: Table S3–S4.

### Multimodal imaging preprocessing

Multimodal brain imaging of resting-state fMRI and sMRI was available from each participant who met inclusion criteria. Detailed image acquisition parameters can be found in the ABIDE website https://fcon_1000.projects.nitrc.org/indi/abide/abide_I.html for ABIDE I and https://.fcon_1000.projects.nitrc.org/indi/abide/abide_II.html for ABIDE II.

The fMRI data were preprocessed using statistical parametric mapping (SPM12, https://www.fil.ion.ucl.ac.uk/spm/) in the MATLAB 2019 environment. We performed rigid body motion correction using SPM to correct subject head motion, followed by the slice-timing correction to account for timing difference in slice acquisition. The fMRI data were subsequently warped into the standard Montreal Neurological Institute (MNI) space using an echo planar imaging (EPI) template and were resampled to 3 × 3 × 3 mm^3^ isotropic voxels. The resampled fMRI images were further smoothed using a Gaussian kernel with a full width at half maximum (FWHM) = 6 mm. Then for each voxel, six rigid body head motion parameters, white matter (WM) signals, and cerebrospinal fluid (CSF) signals were regressed out using linear regression. Finally, to calculate fALFF [34], the sum of the spectral amplitude values in the 0.01–0.08 Hz low-frequency power range was divided by the sum of the amplitudes over the entire detectable power spectrum (range: 0–0.25 Hz). Regarding head motion, we removed outlier datasets with mean FD exceeding 1 mm, as well as head motion exceeding 2.0 mm of maximal translation (in any direction of *x*, *y* or *z*) or 1.0° of maximal rotation throughout the course of scanning. There is no group difference of mean FD among Asperger’s, PDD-NOS and Autistic (Table 1) as well as between TDC group (Additional file 1: Table S3–S4). Furthermore, the fusion analysis was conducted on the spatial maps of fALFF, but not the functional connectivity related feature, which is affected by head motion. Considering that no group differences exist on (micro) head motions during fMRI preprocessing, we believe micro-motion such as FD is not a major factor affecting the current results.

The structural data T1 images were preprocessed through an automated pipeline. Tissue classification, bias correction, image registration and spatial normalization were automatically performed using voxel-based morphometry in SPM12, wherein the above steps are integrated into a unified model [39]. Modulated GM segmentations, which produce an estimation of GMV, were then smoothed using a Gaussian kernel with a width of 6 mm.

Next, each modality was reshaped into a feature matrix with columns representing voxels and rows representing subjects. Since there were group differences among Asperger’s, PDD-NOS and Autistic subgroups in age, handedness and intelligence (Table 1), we regressed out age, handedness, intelligence (full scale IQ) and acquisition site from fALFF and GM data prior to the fusion analysis. Finally, the obtained feature matrices were normalized to have the same average sum of squares (computed across all subjects and all voxels for each modality) to ensure all modalities had the same range of values.

### Study design

**Fig. 1:**
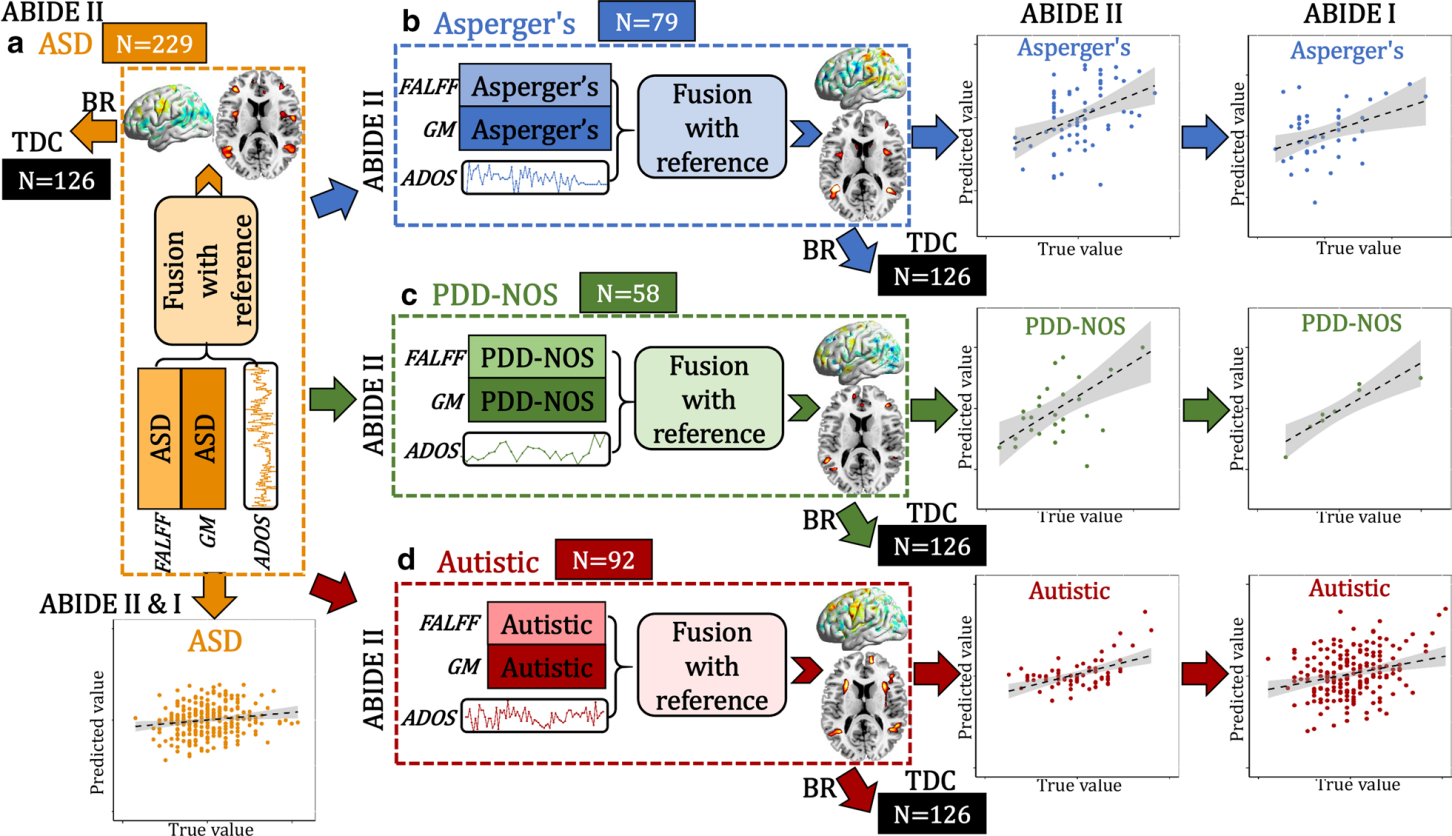
Flowchart of the study design. ADOS composite scores were used as a reference to guide a two-way fALFF-GM fusion for (a, orange) the whole ASD group and then the ASD subgroups [(b, blue) Asperger’s, (c, green) PDD-NOS and (d, red) Autistic] to identify ASD and subtype-related multimodal brain networks associated with ADOS. Then, the identified brain maps were back-reconstructed (BR) on the same control group (TDC). Finally, the identified multimodal patterns were used to train symptom prediction models in each subtype using the ABIDE II data and then cross-validated using the ABIDE I data

**Fig. 2:**
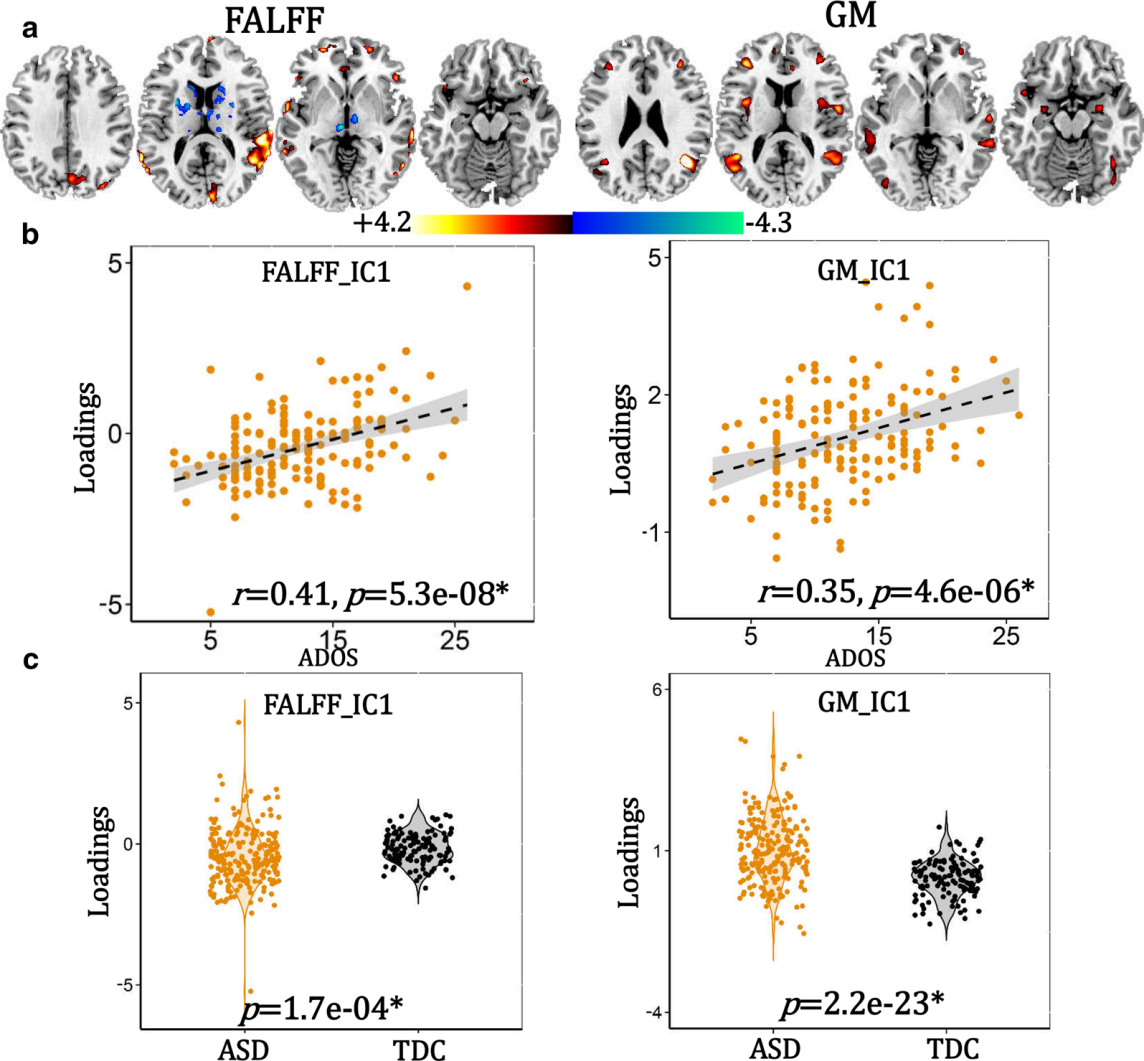
The identified ADOS-associated joint component in ASD. **a** The spatial maps are visualized at |*Z*|> 2 thresholds, where the red regions mean positive fALFF or GMV, and the blue areas indicate negative fALFF or GMV. **b** Correlation between loadings of the identified components and ADOS. *Signifies FDR corrected. **c** Group differences between ASD and TDC of the loading parameters

According to the four goals stated in the introduction, we performed a systematic, data-driven analysis as designed in Fig. 1. Specifically, (1) ADOS composite scores for individuals with ASD were used as a reference to guide a two-way MRI (fALFF + GMV) fusion analysis for the whole ASD (Fig. 1a) sample, and then for each subtype that within it [Asperger’s (Fig. 1b), PDD-NOS (Fig. 1c) and Autistic (Fig. 1d)]; (2) back-reconstruction was performed to verify whether there are group differences between ASD/subtypes and TDCs, of the identified multimodal components; (3) common and unique multimodal brain networks were identified; and (4) multivariate linear regression was performed to cross-validate the predictability of the identified multimodal features in predicting ADOS and SRS scores of ASD and subtypes in an independent sample (ABIDE I).

Specifically, the preprocessed multimodal MRI features were jointly analyzed by a fusion-with-reference model called “MCCAR + jICA” (https://trendscenter.org/software/fit/, multi-site canonical correlation analysis with reference + joint independent component analysis) [40], a data-driven analysis for identifying targeted brain regions associated with the overall ADOS scores. Assume that there are *n* multimodal datasets ${\varvec{X}}_{k}$, and each is a linear mixture of components ${\varvec{S}}_{k}$ with a nonsingular mixing matrix ${\varvec{A}}_{k} .$ $k = 1,2, \ldots ,n$, denotes the modality. Thus,

1 $${\varvec{X}}_{k} = {\varvec{A}}_{k} {\varvec{S}}_{k}$$

where ${\varvec{X}}_{k}$ is a subjects-by-voxels feature matrix and ${\varvec{A}}_{k}$ is in the dimension of subjects by number of components ***M***. Loadings in mixing matrix (${\varvec{A}}_{k,m}$, *m* represents the *m*th column of ${\varvec{A}}_{k}$) represent the contribution weight for each subject in the corresponding component (${\varvec{S}}_{m,k}$, *m* represents the *m*th row of ${\varvec{S}}_{k}$). In supervised fusion, MCCAR + jICA imposes an additional constraint to maximize not only the covariations among mixing matrices of each modality, but also the column-wise correlations between ${\varvec{A}}_{k}$ and the reference signal, as shown in Eq. (2).

2 $$\max \mathop \sum \limits_{k,j = 1}^{2} \left\{ {{\text{corr}}\left( {A_{k} ,A_{j} } \right)_{2}^{2} + 2\lambda \cdot {\text{corr}}\left( {A_{k} ,{\text{ref}}} \right)_{2}^{2} } \right\}$$

where ${\text{ref}}$ is an $N \times 1$ vector, denoting the interested clinical measure (i.e., ADOS), $N$ represents number of subjects. Comparing with unsupervised multimodal fusion methods, *e.g*., MCCA, jICA, MCCAR + jICA is more goal-directed [41] by taking advantage of a prior (interested clinical measure, ADOS) to guide the fusion analysis. Thus, it can simultaneously maximize the inter-modality covariation and correlations of certain imaging components with ADOS. After optimization of Eq. (2), we can obtain the potential target components $\user2{ S}_{i}$ that are correlated with ADOS in each modality. Therefore, by incorporating prior information, MCCAR + jICA enables identification of a joint multimodal component(s) that has robust correlations with referred measures (ADOS), which may not be detected by a blind N-way multimodal fusion approaches [42]. Subject-wise total ADOS scores were used as a reference to jointly decompose fALFF and GM volume to investigate ADOS-associated fALFF-GM covarying multimodal patterns for all ASD and its subtypes. As a result, a joint multimodal component(s) which correlated with total ADOS scores are identified. To generate comparable components among ASD and its three subgroups, we used the same component number (IC = 30) for ASD and each of the three subtypes’ fusion analysis. To establish whether there is group difference of the identified multimodal patterns between patients and TDCs, back-reconstruction of ASD/subtype-related brain maps to the TDCs was performed based on the linear projection model as in Eq. (3).

3 $$\begin{gathered} X_{{{\text{ASD}},{\text{k}}}} = A_{{{\text{ASD}},{\text{k}}}} \times S_{{{\text{ASD}},{\text{k}}}} \hfill \\ A_{{{\text{TDC}},{\text{k}}}} = X_{{{\text{TDC}},{\text{k}}}} \times \left( {S_{{{\text{ASD}},{\text{k}}}} } \right)^{ - 1} \quad k = {1},{ 2} \hfill \\ \end{gathered}$$

where $S_{{{\text{ASD}},{\text{k}}}}$ and $A_{{{\text{ASD}},{\text{k}}}}$ denote the brain components and the corresponding mixing matrix derived by MCCAR + jICA for the whole ASD group. $X_{{{\text{ASD}},{\text{k}}}}$ and $X_{{{\text{TDC}},{\text{k}}}}$ represent preprocessed imaging feature matrixes for ASD and TDC groups separately, and *k* represents the modality. The spatial maps of ASD ($S_{{{\text{ASD}},{\text{k}}}}$) were used to estimate the mixing matrix of the control group ($A_{{{\text{TDC}},{\text{k}}}}$) based on Eq. (3). The same back-reconstruction was performed on ASD and its subtypes separately. Finally, the identified multimodal patterns were used to train symptom prediction models in each subtype using the ABIDE II data and then cross-validated using the ABIDE I data.

## Results

### ASD-related multimodal patterns

ASD-related joint components were identified (Fig. 2), which correlated with total ADOS scores (fALFF: *r* = 0.41, *p* = 5.3e−08; GM: *r* = 0.35, *p* = 4.6e−06), which are false discovery rate correction (FDR) corrected. The positive/negative brain regions (red/blue) indicate positive/negative correlation with ADOS scores in fALFF or GMV. ASD-related multimodal patterns include positive brain fALFF in dorsolateral prefrontal cortex (DLPFC, BA9 and BA46, including the superior/middle frontal gyrus), inferior frontal gyrus (Broca’s area, BA45), superior/middle temporal gyrus (SM_TG), insula, lingual gyrus and angular gyrus, and negative fALFF in thalamus and caudate, accompanied with positive GM volume in DLPFC, SM_TG, superior/inferior parietal lobule, angular gyrus, lingual gyrus, fusiform gyrus, insula and amygdala, as summarized in Additional file 1: Table S5.

### Subtype-related multimodal patterns

Asperger’s, PDD-NOS and Autistic-related joint components were also identified (Additional file 1: Fig S1–S3). The identified brain regions are summarized in Additional file 1: Table S6–S8 for fALFF and GM (Talairach labels), respectively. For the subgroup diagnosed as Asperger’s, ADOS scores are positively correlated with brain fALFF in DLPFC, SM_TG, superior/inferior parietal cortex and angular gyrus, and negatively associated with fALFF in fusiform gyrus, lingual gyrus, putamen and parahippocampus, accompanied with positive GM volume in DLPFC, SM_TG, inferior parietal cortex, insula and angular gyrus. For PDD-NOS, ADOS scores are positively associated with brain fALFF in DLPFC, SM_TG, superior/inferior parietal cortex, angular gyrus, lingual gyrus and fusiform, and negatively related with fALFF in anterior cingulate cortex (ACC) and caudate, accompanied by positive GM volume in DLPFC, ST_TG, Broca’s area, lingual gyrus and fusiform. For autistic group, ADOS scores are positively associated with higher fALFF in DLPFC, SM_TG, inferior parietal cortex, angular gyrus, and lingual and negatively correlated with fALFF in caudate, thalamus and amygdala, accompanied with positive GM volume in DLPFC, ST_TG, Broca’s area, superior/inferior parietal cortex, angular gyrus, fusiform and lingual cortex. The partial correlations between subtype components and ADOS remain significant even after regressing out FD (Additional file 1: Table S9), site and TR (Additional file 1: Table S10).

### Group difference between ASD/subtype and TDCs

The identified ASD and subtype-specific components preserve group differences between ASD and controls, and between each subtype and controls (Fig. 2c and Additional file 1: Fig S1c–S3c). These group differences remain significant even after FIQ, age and handedness were regressed out (Additional file 1: Table S11).

### Common and unique patterns among three subtypes

**Fig. 3:**
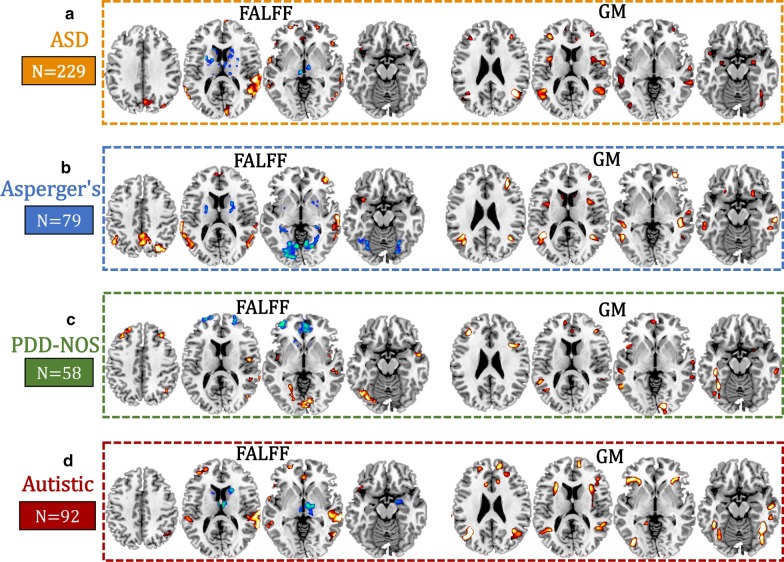
Comparison of multimodal patterns (the same slices) among **a** the whole ASD (*n* = 229), **b** Asperger’s (*n* = 79), **c** PDD-NOS (*n* = 58) and **d** Autistic (*n* = 92) subgroups. Each subtype-related patterns are correlated with ADOS (details can be found in Additional file 1: Fig. S1–S3)

Subtype-specific multimodal patterns on the same slices among the Asperger’s, PDD-NOS and Autistic subgroups were compared to the overall ASD multimodal patterns (Fig. 3). Positive fALFF in DLPFC and SM_TG accompanied with positive GM volume in DLPFC and SM_TG were the common functional–structural covaried patterns seen in ASD and also among Asperger’s, PDD-NOS and Autistic subtypes. These are the shared core multimodal attributes. In contrast, the main differences when comparing among subtypes are negative functional subcortices. Specifically, negative subcortical brain fALFF, including putamen and parahippocampus, are unique for the Asperger’s subtype; negative fALFF in ACC is unique for the PDD-NOS subtype; and negative amygdala, caudate and thalamus are unique to the Autistic subtype. Moreover, when comparing fALFF and GM patterns, we found that GMV increase is more consistent than fALFF abnormalities over the three subgroups, where only subtle differences exist. For example, although positive GMV in the insular cortex was identified for all the three subtypes, they occur in different parts of the insula (anterior insula for Autistic and PDD-NOS; middle insula for Asperger’s).

### Different associations with ADOS subdomains

We also assessed the correlation between the identified subtype-related multimodal brain networks with subdomain scores of the ADOS (Table 2). Results show that Asperger’s-related neural pattern is associated with scores from the ADOS social interaction subdomain only, as might be expected based on this diagnostic subtype’s diagnostic criteria. PDD-NOS-related pattern is correlated with both social communication and social interaction subdomains, while Autistic neural patterns are correlated with all three subdomains (social communication, social interaction and stereotyped behaviors). Social interaction is the common subdomain that associates with the Asperger’s, PDD-NOS and Autistic brain components. As for age and motion, we found that the identified subtype-related patterns were uncorrelated with neither age nor mean FD (Additional file 1: Table S12).

**Table 2:** Correlation between subtype-related components with subdomains of ADOS

	Social communication	Social interaction	Stereotyped behaviors and restricted interest
Asperger’s			
fMRI_IC	*r* = 0.30, *p* = 0.02	*r* = 0.46, *p* = 1.2e−04*	*r* = 0.38, *p* = 0.002
sMRI_IC	*r* = 0.27, *p* = 0.03	*r* = 0.57, *p* = 8.1e−07*	*r* = 0.40, *p* = 0.001
PDD-NOS			
fMRI_IC	*r* = 0.70, *p* = 6.0e−05*	*r* = 0.72, *p* = 2.3e−05*	*r* = 0.16, *p* = 0.44
sMRI_IC	*r* = 0.84, *p* = 3.9e−08*	*r* = 0.83, *p* = 6.2e−08*	*r* = 0.22, *p* = 0.27
Autistic			
fMRI_IC	*r* = 0.43, *p* = 1.6e−04*	*r* = 0.41, *p* = 3.4e-04*	*r* = 0.41, *p* = 3.2e-04*
sMRI_IC	*r* = 0.36, *p* = 0.002	*r* = 0.46, *p* = 3.5e-05*	*r* = 0.32, *p* = 0.006

*Signifies FDR correction for multiple comparisons

### Cross-cohort prediction

To verify the predictability [43, 44] of these specific multimodal brain features identified within ASD and the subtypes, the extracted ROIs (positive and negative brain networks in fMRI_IC, and positive brain networks in sMRI_IC, details can be found in Supplementary “Predictive feature extraction” section) were used as regressors to predict multiple symptom scores (ADOS and SRS) using an independent sample (ABIDE I) for cross-validation. Based on the following Eq. (4), a multiple linear regression model was trained on ABIDE II.

4 $${\text{Symptom scores}} = \beta_{0} + {\text{fMRI}}\_{\text{positive}} \times \beta_{1} + {\text{fMRI}}\_{\text{negative}} \times \beta_{2} + {\text{sMRI}} \times \beta_{3}$$

The predictive accuracy is measured by the correlation between the estimated cognitive scores and its true values, as well as the normalized root mean squared prediction error (NRMSE). The NRMSE is a frequently used measure of the differences between values predicted by a model or an estimator and the values observed. The RMSE of predicted values $\widetilde{{y_{n} }}$ for subject *n* of a regression’s dependent variable $y_{n}$ with variables observed over *N* subjects, is computed for *N* different predictions as the square root of the mean of the squares of the deviations:

5 $${\text{RMSE}} = \sqrt[2]{{\frac{{\mathop \sum \nolimits_{n = 1}^{N} \left( {\widetilde{{y_{n} }} - y_{n} } \right)^{2} }}{N}}}$$

6 $${\text{NRMSE}} = \frac{{{\text{RMSE}}}}{{y_{\max } - y_{\min } }}$$

**Fig. 4:**
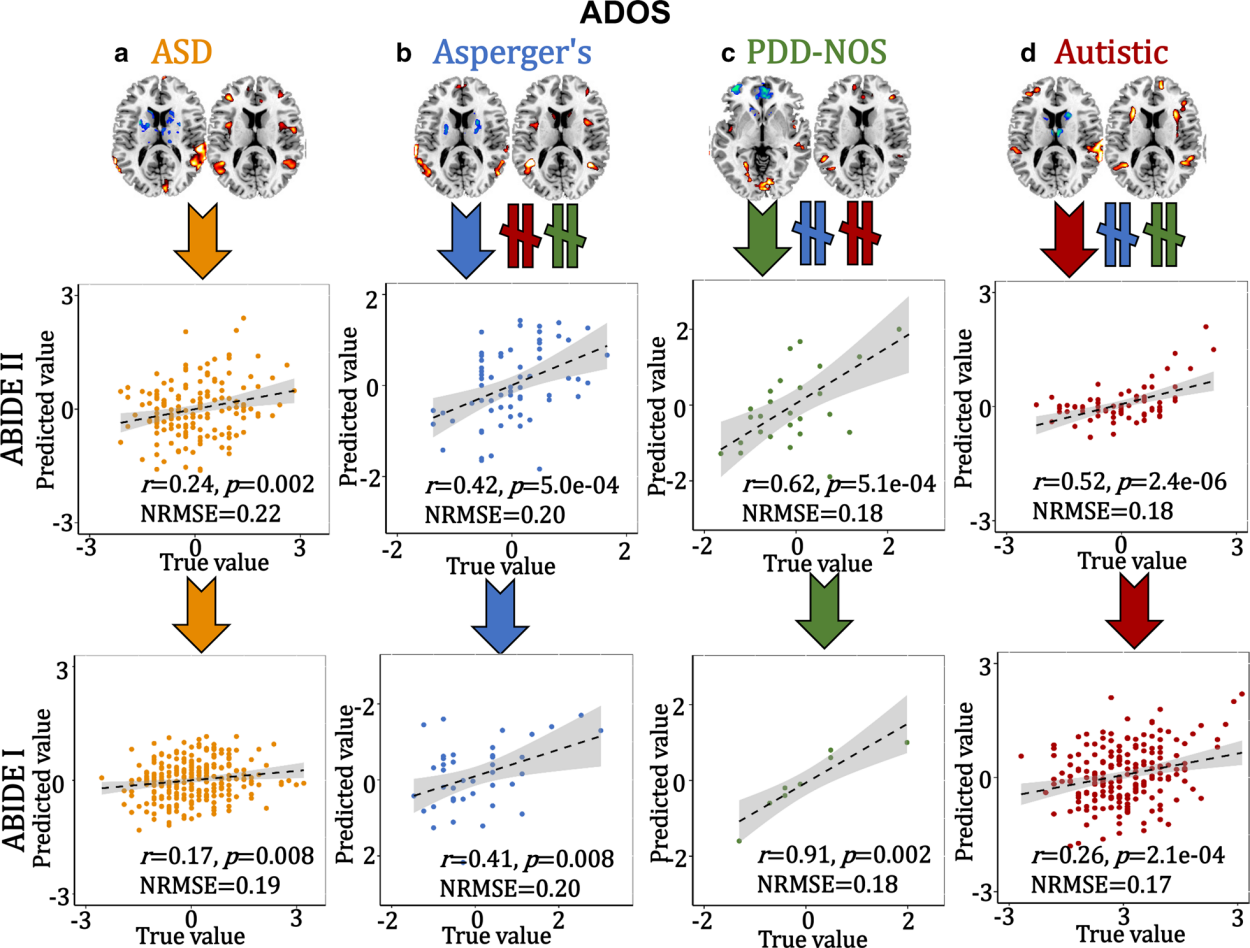
Prediction analysis on ADOS scores. The identified ASD (**a**), Asperger’s (**b**), PDD-NOS (**c**) and Autistic (**d**)-related brain areas (positive and negative brain networks in fALFF plus positive brain areas in GM, 3-dimensional features) were used as features to train a multiple linear regression model in ABIDE II cohort. Then, the brain areas and the prediction models were generalized to predict the corresponding groups’ ADOS and SRS scores in an independent ABIDE I cohort. Arrows (orange, blue, green and red represent ASD, Asperger’s, PDD-NOS and Autistic groups, respectively) mean the features are predictive for the corresponding group. “$\ne$” denotes the features from this subgroup are not predictable for the other two subgroups

**Fig. 5:**
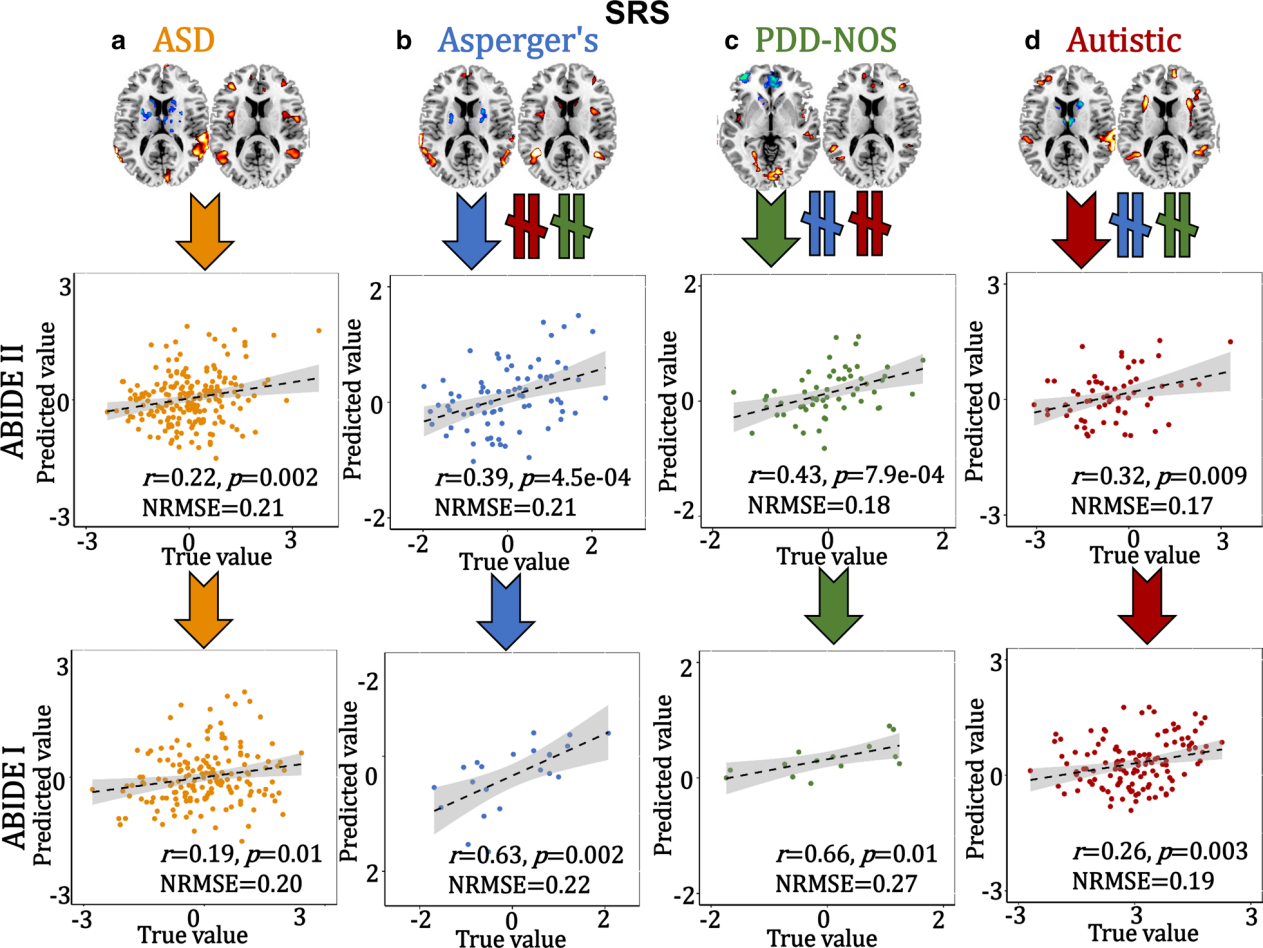
Prediction analysis on SRS scores. The identified ASD (**a**), Asperger’s (**b**), PDD-NOS (**c**) and Autistic (**d**)-related brain areas (positive and negative brain networks in fALFF plus positive brain areas in GM, 3-dimensional features) were used as features to train a multiple linear regression model in ABIDE II cohort. Then, the brain areas and the prediction models were generalized to predict the corresponding groups’ ADOS and SRS scores in an independent ABIDE I cohort. Arrows (orange, blue, green and red represent ASD, Asperger’s, PDD-NOS and Autistic groups, respectively) mean the features are predictive for the corresponding group. “$\ne$” denotes the features from this subgroup are not predictable for the other two subgroups

The 3-dimensional features were predictable for ADOS and SRS (SRS was not correlated with ADOS, *r* = 0.07, *p* = 0.4) scores for ABIDE II and the same prediction models can be generalized to predict ADOS and SRS scores for the corresponding subgroup from ABIDE I, but not for the other groups (Figs. 4, 5).

### Reproducibility across sites

There are studies showing that the ASD case–control differences of resting state functional connectivity were inconsistent across sites regardless of choice of denoising strategies [45]. However, our current study changes from the case–control model to an ASD subtype dimensional model, and we used fALFF but not the functional connectivity in the fusion analysis. To show the reproducibility, we used two different ways to discuss replication across sites. The first one is that we performed leave-one-site-out analysis (details can be found in Additional file 1: “Reproducibility” section) in the ADOS-guided fALFF and GMV fusion on the whole ASD group to check whether the site effect will contribute to current ASD pattern. Here, we present the most frequently occurring voxels (those which occur more than 70% of sites, i.e., 6 sites among 9) associated with ADOS among these 9 repeated leave-one-site-out analysis, as shown in Additional file 1: Fig. S4b. Note that the overlapped model of spatial patterns is highly similar with the ASD pattern (all sites included). This result shows that the altered covarying patterns of fALFF-GMV of DLPFC, superior–middle temporal gyrus and insula in ASD group are consistent across 70% sites in ABIDE II.

The second one is that we performed linear projection (details can be found in Additional file 1: “Reproducibility” section) of the identified subtype-related components from ABIDE II to the corresponding subgroups in ABIDE I. Results (Additional file 1: Table S13) show that the linkage between subtype-related components and ADOS detected in ABIDE II can be replicated in ABIDE I age-matched cohort. Considering (1) site was regressed out from fALFF and GMV prior to fusion analysis; (2) partial correlations between subtype’s components and ADOS remain significant after regressing out site (Additional file 1: Table S10); (3) the most frequently occurring voxels (those which occur more than 70% across sites) are similar with the original ASD pattern (Additional file 1: Fig. S4); (4) the linkage between subtype components and ADOS by projecting from ABIDE II to ABIDE I remains significant (Additional file 1: Table S13); we conclude that the site effect would not be a main factor affecting the current results.

## Discussion

**Fig. 6:**
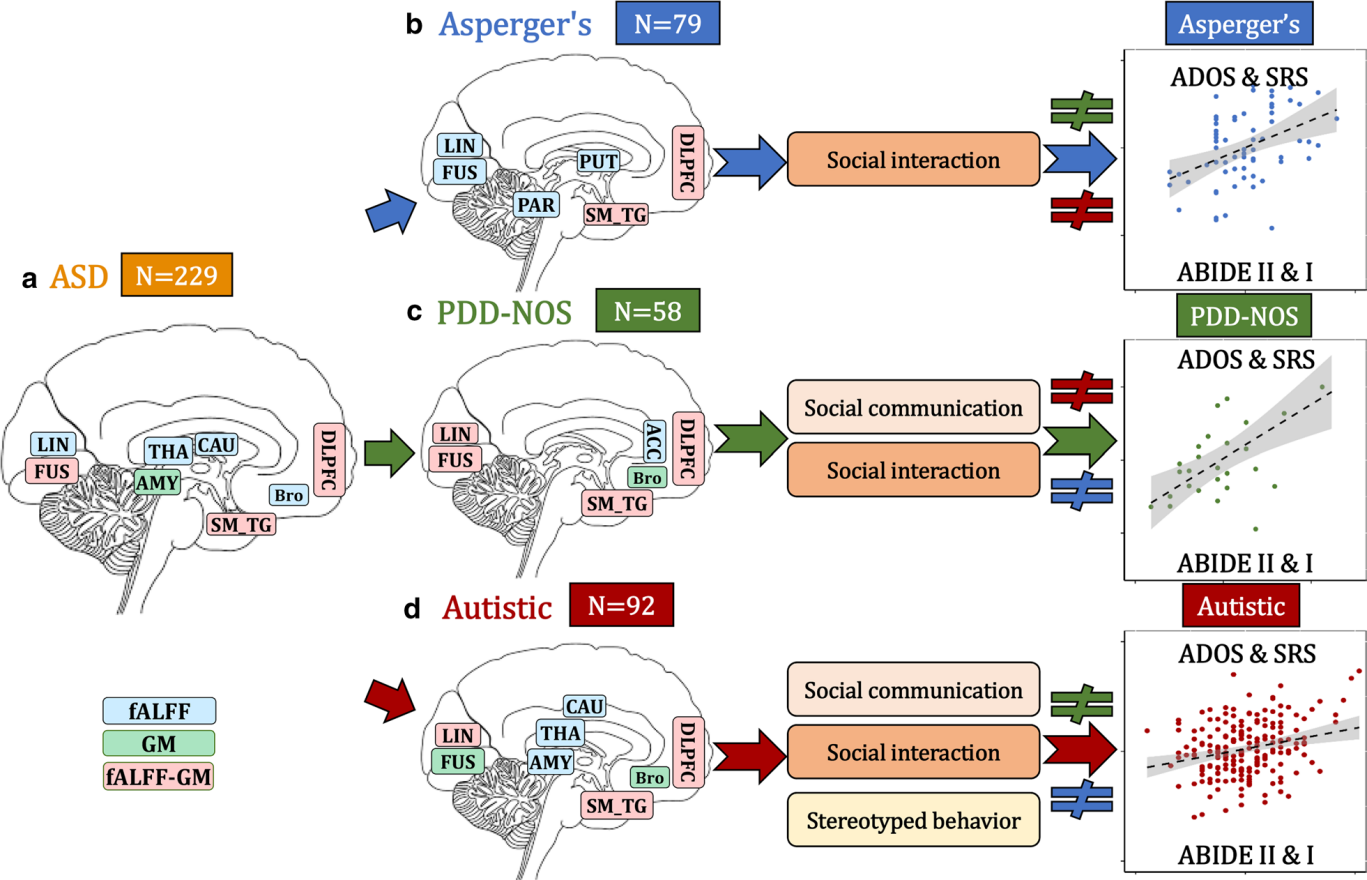
Summary on ASD and its subtypes related fALFF-GM covarying patterns: ASD (**a** orange), Asperger’s (**b** blue), PDD-NOS (**c** green) and Autistic (**d** red). The DLPFC and SM_TG are the common functional–structural covarying cortical brain areas among ASD and its related Asperger’s, PDD-NOS and Autistic subgroups. Fusiform and lingual gyrus are also the common brain areas for the three subtypes, but with different modalities. The main differences comparing Asperger’s, PDD-NOS and Autistic are the negative functional subcortical brain areas, including negative putamen–parahippocampus that is unique to Asperger’s subgroup; negative ACC that is unique to PDD-NOS subgroup and negative thalamus–amygdala–caudate that is unique to the Autistic subgroup. Broca’s area was identified for Autistic and PDD-NOS subgroups, but not for Asperger’s. Each subtype-related pattern is correlated differentially with ADOS subdomains, and these features only predict the corresponding groups but not others. Arrows in the right column mean the features are predictive for the corresponding subgroup. “$\ne$” denotes the features from this subgroup are not predictable for the other two subgroups. DLPFC is dorsolateral prefrontal cortex; SM_TG is superior and middle temporal gyrus; THA is thalamus; AMY is amygdala; CAU is caudate; FUS is fusiform; PAR is parahippocampus; PUT is putamen; ACC is anterior cingulate cortex

This study makes key contributions to the understanding of brain imaging heterogeneity in ASD by identifying both common and unique patterns from multimodal imaging perspectives. As summarized in Fig. 6, our investigation demonstrated the following results. First, DLPFC and SM_TG are common functional–structural covarying cortical brain areas shared among ASD and its subgroups. The main differences comparing Asperger’s, PDD-NOS and Autistic are primarily the negative functional subcortical brain areas. Second, each subtype-related pattern is correlated differentially with ADOS subdomains, with social interaction as the common subdomain. Third, these subtype-related features can only predict the corresponding subgroups, but not the others.

A major finding is the identification of the shared fALFF and GM covaried patterns within the ASD sample and its subgroups, suggestive of core ASD neural–behavioral characteristics across ASD severity dimension. The identified alterations in DLPFC and SM_TG are the most consistent findings related to many of the behavioral deficits observed in individuals with ASD, such as receptive language, social cognition, joint attention, action observation and empathy [10, 46]. DLPFC and SM_TG are also regions where the strongest case–control differences between ASD and TDCs are observed [10]. Fusiform, which is reported to be related with impaired processing of faces in ASD [47, 48], is also the common brain area for ASD and its subtypes, but different subtypes are related with different modalities. These common brain areas would appear to partly support the conceptual merger of these DSM-IV subtypes into the DSM-5 ASD framework. Note that the identified common multimodal cortical brain regions across all subtypes are heavily involved in social and communication-related brain regions [49, 50]. Broca's area dysfunction, which is involved in the production and expression of language, was identified for Autistic and PDD-NOS subgroups, but not for Asperger’s. This is consistent with the traditional concept that the Asperger’s subtype is differentiated from the other subgroups based on their more intact oral language capabilities.

Outside of the core neural–behavioral characteristics shared by all subgroups, the main differences when comparing Asperger’s, PDD-NOS and Autistic subgroups are primarily within functional subcortical brain areas, including negative putamen–parahippocampus unique to Asperger’s; negative ACC unique to PDD-NOS and negative thalamus–amygdala–caudate unique to the Autistic subgroup. These results highlight the very different negative fALFF functioning differentiating ASD subgroups with almost no overlap of their unique localized findings of fMRI measure. These unique differences in fMRI functioning may underlie different mechanisms and the known levels of severity among ASD subgroups. Dysfunction of putamen found in high-functioning adults with ASD [51] and decreased inter- and intra-hemispheric functional connectivity density in parahippocampus [52] may underlie the pathologies of ASD. An animal study has shown that ACC (identified in Autistic and PDD-NOS subgroups in our results) has a role in the regulation of social behavior in mice and indicates that ACC dysfunction may be involved in social impairments in ASD [53]. Note that ACC in fALFF was neutralized in the whole ASD sample due to the negative fALFF in PDD-NOS and positive fALFF findings in Autistic subtype, which implies that different functions of ACC may play in PDD-NOS and Autistic subtypes. The Autistic subtype and the whole ASD sample both showed thalamus–amygdala–caudate findings, which may relate with the stereotyped behaviors and restricted interest in Autistic subtypes (Table 2). This is consistent with findings that the Autistic subtype has been shown to have the highest levels of concordance when diagnosed using either the DSM-IV or DSM-5 systems [54].

Another interesting finding is that each subtype’s unique multimodal patterns are correlated differently with different ADOS subdomains, with the expectation that the social interaction subdomain is the common dysfunction across the three subtypes. This is also consistent with the known core diagnostic criteria of deficits in social interaction in each subtype. Asperger’s is primarily associated with difficulties in the social communication domain, consistent with this subtypes primary defining attributes; PDD-NOS showed a pattern associated with difficulties in the social communication and interaction domains, generally somewhat consistent with its traditional attributes, although they can have some atypical/stereotype behaviors at times; while the Autistic subtype-related patterns were associated with difficulties across all the three ADOS subdomains, consistent with having the most severe combination of ASD attributes, and frequently the most severe combination of all the ASD attributes.

The DSM-5 conceptually combined the earlier DSM-IV diagnostic subtypes (Asperger’s, PDD-NOS and Autistic disorder) into a broader and more heterogeneous disorder of ASD. This was due to the historically poor reliability in how subtypes were diagnosed in individuals across raters, and their questionable validity, but it still emphasized that ASD can range from very mild to more severe symptomatology, consistent with our sample and results. So although the DSM-5 provides for the diagnosis of ASD in those patients who had been diagnosed previously using the DSM-IV and given a diagnosis of Autistic, Asperger’s or PDD-NOS, research that has studied the two system’s diagnostic concordance suggests good overlap for the Autistic subtype and ASD, but lower for the PDD-NOS and Asperger’s subtypes [54]. Given that the current ASD samples were derived by merging these subtype samples, originally diagnosed using the DSM-IV criteria, we were not able to independently re-diagnose them nor validate them using the current DSM-5 criteria. We expect there to be a number of cases in these subtype samples who may not currently meet the revised DSM-5 ASD criteria, thus adding noise to our comparisons. At the same time, we cross-validated these results across an independent sample, adding to their generalizability strength to these findings. We believe that these findings can add to the debates regarding the differences between these different diagnostic frameworks and their underlying conceptualizations. This is particularly due to the findings of both common and distinctive subtype-specific areas of functional–structural dysfunction that correlated with the common and sometimes unique behavioral attributes of ASD and its historical subtypes. Thus, we emphasize that there are both common (consistent with the DSM-5 ASD conceptualization) and possibly unique (consistent with the DSM-IV subtype conceptualization) findings of functional–structural dysfunction among Asperger’s, PDD-NOS and Autistic disorder.

## Limitations

A potential limitation is that the ABIDE data were collected from multiple sites. The lack of standardization across MRI acquisitions, inclusion criteria, TR, eyes-open vs. eyes-closed status and clinical assessments should be considered. For example, the eyes-open status (eyes-open percentages are 86%, 97%, 100% for Asperger’s, PDD-NOS and Autistic, respectively) would confound the current findings, introducing the regional differences at lingual and fusiform areas between Asperger’s and PDD-NOS. However, it can also be considered as an advantage, given that even with this level of heterogeneity in the diagnostic and imaging methods, the prediction models can be generalized from ABIDE II to ABIDE I on both ADOS and SRS scores. Another limitation is that this male-based sample cannot be generalized to female ASD population. Note that there is a slight difference in the distribution of ADOS items across subdomains used to generate the total ADOS symptom scores despite equal maximal total scores across four modules of the ADOS. However, the majority of the data in these total ADOS scores (97% for ABIDE II and 99% for ABIDE I) of ASD subjects were based on ADOS-G modules 3 or 4, which have the same number of items across the 3 different domains. Although this study used static brain function (fALFF) approaches, dynamic functional network connectivity matrices [55, 56] can also be used to capture both temporal and spatial co-alterations in a future study, which could provide an even richer understanding of both the core and unique aspects of ASD on temporal perspective. Such an analysis pipeline could also be directly applied to study additional populations of interest which also show significant heterogeneity and diagnostic debated (i.e. mild cognitive impairment and Alzheimer’s dementia) [57].

## Conclusions

To the best of our knowledge, this is the first attempt to evaluate ASD subtype heterogeneity under a multimodal fusion and cross-cohort prediction framework, and such heterogeneity is more prevalent in fMRI than sMRI. We not only identified functional–structural DLPFC and SM_TG as the common brain regions across Asperger’s, PDD-NOS and Autistic, but also confirmed that the main differences were negative functional subcortical areas. The predictability of the identified subtype-related patterns used in predicting symptom scores in an independent sample (ABIDE I) verifies the uniqueness of the different neural features of these traditional subtypes. According to these results, we conclude that although ASD has a common neural basis that is consistent with the core deficits involved primarily in social interaction, each of the different traditional ASD subtypes is strongly linked to unique multimodal covarying brain systems. The identified core and unique subtype-related patterns may help us to better understand the underlying mechanisms involved in the behavioral heterogeneity, particularly as related to the range of severity in ASD from a multimodal brain imaging perspective.

## Supplementary information

**Additional file 1: Figure S1**. The identified ADOS-associated joint component in Asperger’s subgroup. **Figure S2**. The identified ADOS-associated joint component in PDD-NOS subgroup. **Figure S3**. The identified ADOS-associated joint component in Autistic subgroup. **Figure S4**. Reproducibility across sites by leave-one-site-out. **Table S1**. Sites information for ABIDE II and ABIDE I for ASD participants. **Table S2**. Correlation between ADOS and other clinical scores. **Table S3**. Demographic and clinical information comparing patients and controls. **Table S4**. Group difference with TDC of some clinical measures in ABIDEII. **Table S5**. Anatomical information of ASD related multimodal brain areas associated with ADOS. **Table S6**. Anatomical information of Asperger’s related multimodal brain areas associated with ADOS. **Table S7**. Anatomical information of PDD-NOS related multimodal brain areas associated with ADOS. **Table S8**. Anatomical information of Autistic related multimodal brain areas associated with ADOS. **Table S9**. The partial correlations between components’ loadings and ADOS after regressing out FD. **Table S10**. The partial correlations between components and ADOS after regressing out both TR and site. **Table S11**. Group difference with TDC of the identified component after controlling age, handedness, FIQ. **Table S12**. Correlation between subtype-related components with age and the mean FD. None of them is significant. **Table S13**. Correlation between ADOS and subtype components by projecting from ABIDE II to ABIDE I cohort.

## Data Availability

The supervised fusion code has been released and integrated in the Fusion ICA Toolbox (FIT, https://trendscenter.org/software/fit), which can be downloaded and used directly by users worldwide. The multimodal MRI data used in this study can be accessed upon application from ABIDE consortium.

## Abbreviations

ASD: Autism spectrum disorder
ADOS: Autism Diagnostic Observation Schedule
PDD-NOS: Pervasive developmental disorder-not otherwise specified
fMRI: Functional magnetic resonance imaging
sMRI: Structural MRI
fALFF: Fractional amplitude of low-frequency fluctuations
ABIDE: Autism Brain Imaging Data Exchange
GMV: Gray matter volume
SRS: Social Responsiveness Scale
TDC: Typically developing controls
FD: Framewise displacements
BR: Back-reconstructed
MCCAR + jICA: Multi-site canonical correlation analysis with reference + joint independent component analysis
DLPFC: Dorsolateral prefrontal cortex
SM_TG: Superior/middle temporal gyrus
ACC: Anterior cingulate cortex
