# Supplementary material for: Common and unique multimodal covarying patterns in autism spectrum disorder subtypes
Source: Mol Autism. 2020 Nov 18;11:90. doi: 10.1186/s13229-020-00397-4 (PMC7673101; doi:10.1186/s13229-020-00397-4)
Supplement: Supplementary file 1 — Additional file 1: Figure S1. The identified ADOS-associated joint component in Asperger’s subgroup. Figure S2. The identified ADOS-associated joint component in PDD-NOS subgroup. Figure S3. The identified ADOS-associated joint component in Autistic subgroup. Figure S4. Reproducibility across sites by leave-one-site-out. Table S1. Sites information for ABIDE II and ABIDE I for ASD participants. Table S2. Correlation between ADOS and other clinical scores. Table S3. Demographic and clinical information comparing patients and controls. Table S4. Group difference with TDC of some clinical measures in ABIDEII. Table S5. Anatomical information of ASD related multimodal brain areas associated with ADOS. Table S6. Anatomical information of Asperger’s related multimodal brain areas associated with ADOS. Table S7. Anatomical information of PDD-NOS related multimodal brain areas associated with ADOS. Table S8. Anatomical information of Autistic related multimodal brain areas associated with ADOS. Table S9. The partial correlations between components’ loadings and ADOS after regressing out FD. Table S10. The partial correlations between components and ADOS after regressing out both TR and site. Table S11. Group difference with TDC of the identified component after controlling age, handedness, FIQ. Table S12. Correlation between subtype-related components with age and the mean FD. None of them is significant. Table S13. Correlation between ADOS and subtype components by projecting from ABIDE II to ABIDE I cohort. [file 13229_2020_397_MOESM1_ESM.pdf]

Supporting information

Table S1. Sites information for ABIDE II and ABIDE I for ASD participants.

ABIDE II (9 sites)	Number of subjects for different subtypes at each site
GU_1	Autistic (*n* = 23); Asperger's (*n* = 13); PDD-NOS (*n* = 6)
IP_1	Asperger's (*n* = 12); Asperger's (*n* = 2); PDD-NOS (*n* = 0)
KKI_1	Asperger's (*n* = 15); Asperger's (*n* = 21); PDD-NOS (*n* = 1)
NYU_1	Autistic (*n* = 12); Asperger's (*n* = 2); PDD-NOS (*n* = 28)
TCD_1	Autistic (*n* = 7); Asperger's (*n* = 14); PDD-NOS (*n* = 0)
UCLA_1	Autistic (*n* = 13); Asperger's (*n* = 0); PDD-NOS (*n* = 0)
USM_1	Autistic (*n* = 10); Asperger's (*n* = 0); PDD-NOS (*n* = 1)
KUL_3	Autistic (*n* = 0); Asperger's (*n* = 27); PDD-NOS (*n* = 1)
NYU_2	Autistic (*n* = 0); Asperger's (*n* = 0); PDD-NOS (*n* = 21)
**ABIDE I (17 sites)**	**Number of subjects for different subtypes at each site**
PITT	Autistic (*n* = 25); Asperger's (*n* = 0); PDD-NOS (*n* = 0)
SDSU	Autistic s (*n* = 2); Asperger's (*n* = 7); PDD-NOS (*n* = 2)
TRINITY	Autistic (*n* = 10); Asperger's (*n* = 7); PDD-NOS (*n* = 7)
UM_1	Autistic (*n* = 39); Asperger's (*n* = 6); PDD-NOS (*n* = 0)
UM_2	Autistic (*n* = 9); Asperger's (*n* = 3); PDD-NOS (*n* = 0)
USM	Autistic (*n* = 53); Asperger's (*n* = 0); PDD-NOS (*n* = 1)
YALE	Autistic (*n* = 4); Asperger's (*n* = 6); PDD-NOS (*n* = 10)
CMU	Autistic (*n* = 8); Asperger's (*n* = 0); PDD-NOS (*n* = 0)
LEUVEN_1	Autistic (*n* = 14); Asperger's (*n* = 0); PDD-NOS (*n* = 0)
LEUVEN_2	Autistic (*n* = 12); Asperger's (*n* = 0); PDD-NOS (*n* = 0)
KKI	Autistic (*n* = 10); Asperger's (*n* = 8); PDD-NOS (*n* = 0)
NYU	Autistic (*n* = 45); Asperger's (*n* = 17); PDD-NOS (*n* = 5)
UCLA_1	Autistic (*n* = 42); Asperger's (*n* = 0); PDD-NOS (*n* = 0)
UCLA_2	Autistic (*n* = 13); Asperger's (*n* = 0); PDD-NOS (*n* = 0)
MAX_MUN	Autistic (*n* = 2); Asperger's (*n* = 14); PDD-NOS (*n* = 0)
CALTECH	Autistic (*n* = 9); Asperger's (*n* = 0); PDD-NOS (*n* = 0)
SBL	Autistic (*n* = 2); Asperger's (*n* = 5); PDD-NOS (*n* = 3)

Table S2. Correlation between ADOS and other clinical scores.

**ABIDE II**	Age	Handedness	FIQ	Mean FD	SRS
ADOS	r=-0.14, p=0.07	r=0.02, p=0.8	r =-0.25, p =0.002	r =-0.02, p =0.8	r=0.07, p =0.4
**ABIDE I**					
ADOS	r=-0.04, p=0.6	r=0.03, p=0.7	r =-0.21, p =0.001	r=0.03, p=0.6	r=0.09, p =0.4

FIQ: full scale IQ. FD: framewise displacements. Although FIQ is correlated with ADOS, we regressed out age, handedness, FIQ and acquisition site from fALFF and GM data prior to the fusion analysis. Spearman correlation instead of Pearson correlation was used to calculate correlation r values between ADOS and handedness.

Table S3. Demographic and clinical information comparing patients and controls.

ABIDE II	Asperger	PDD-NOS	Autistic	TDC	ANOVA
Sample size	n = 79	n = 58	n = 92	n = 126	Na
Age (mean±std)	16.3±6.4	8.3±2.5	12.2±3.8	12.1±5.1	3.4e-18
Gender (M/F)	79/0	58/0	92/0	126/0	Na
Handedness (R/L/M)	67/7/5	36/4/18	75/7/10	109/6/11	0.005
Intelligence (mean±std)	110.4±15.6	104.4±17.5	103.8±20.0	117.5±14.0	1.6e-08
Mean FD (mean±std)	0.1±0.2	0.1±0.2	0.5±0.1	0.1±0.1	0.32

ANOVA column presents the p values for ANOVA test among Asperger's, PDD-NOS, Autistic and TDCs. For handedness (categorical measures), chi-square was applied.

Table S4. Group difference with TDC of some clinical measures in ABIDEII.

	ASD vs.TDC p values	Asperger vs.TDC p values	PDD-NOS vs. TDC p values	Autistic vs. TDC p values
Age	0.39	2.4e-06	1.1e-10	0.9
Handedness	0.03	0.7	0.008	0.2
Intelligence	1.5e-09	0.002	3.9e-06	3.6e-07
Mean FD	0.2	0.2	0.5	0.3

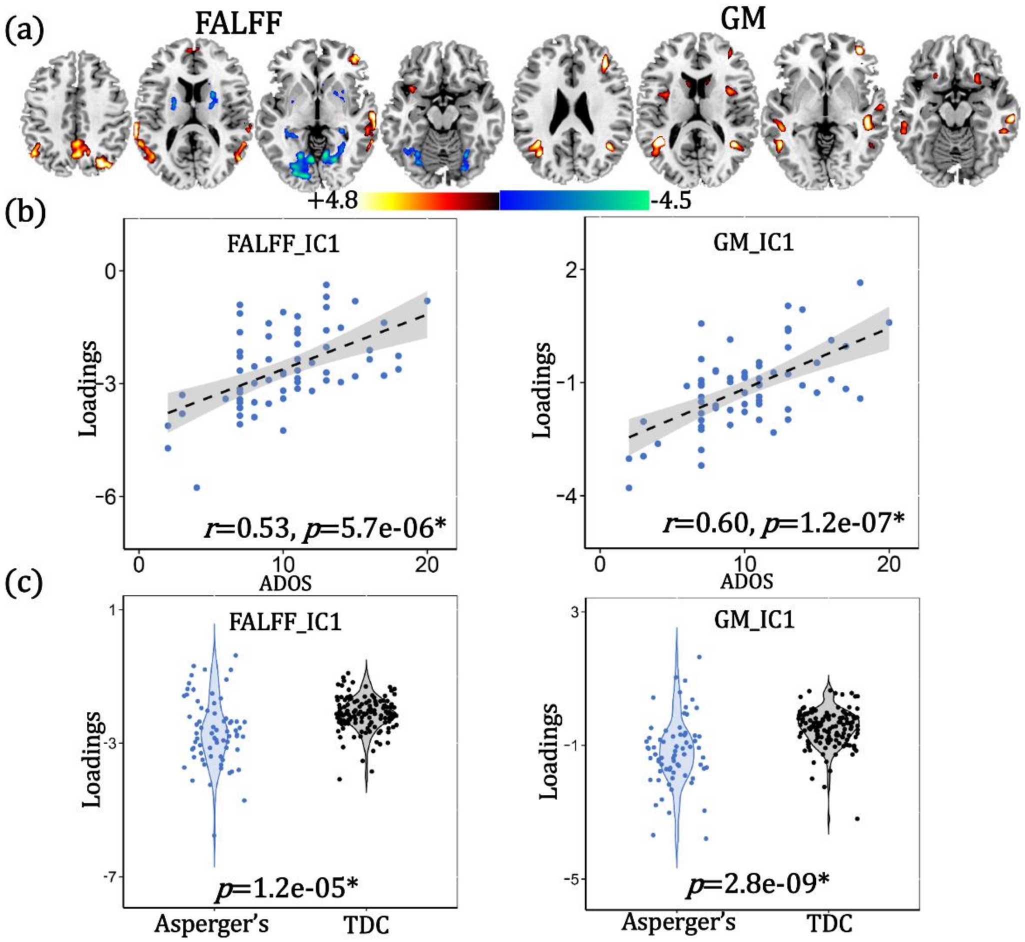

Figure S1. The identified ADOS-associated joint component in Asperger's subgroup. (a) The spatial maps are visualized at |Z|>2 thresholds, where the red regions mean positive fALFF or GMV, and the blue areas indicate negative fALFF or GMV. (b) Correlation between loadings of the identified components and ADOS. (c) Group differences between Asperger's and controls of the loading parameters.

Table S5. Anatomical information of ASD related multimodal brain areas associated with ADOS.

fMRI_IC Area	Brodmann Area	volume (cc)	random effects: Max Value (x, y, z)
Positive			
Superior/Middle Temporal Gyrus	19, 21, 22, 37, 39, 42	1.5/4.8	3.2 (-50, -57, 22)/3.6 (56, -31, 15)
Insula	13	0.1/0.3	2.1 (-56, -37, 21)/3.0 (56, -34, 18)
Superior/Middle/Inferior Frontal Gyrus	6, 10, 11, 45, 46, 47	0.5/1.2	2.4 (-53, 26, 4)/2.5 (53, 26, 1)
Lingual Gyrus	17, 18	0.2/0.5	2.5 (-18, -82, -14)/2.6 (15, -90, 2)
Angular Gyrus	39	0.1/0.1	2.1 (-45, -74, 31)/2.2 (50, -65, 36)
Negative			
Thalamus		0.3/0.3	2.6 (-15, -23, 12)/2.3 (6, -5, 11)
Caudate		0.5/0.4	2.5 (-12, -2, 17)/2.5 (15, -5, 20)
sMRI_IC Area	Brodmann Area	volume (cc)	random effects: Max Value (x, y, z)
Positive			
Superior/Middle Temporal Gyrus	19, 21, 22, 39	1.4/2.7	2.9 (-45, -49, 11)/4.8 (42, -51, 25)
Angular Gyrus	39	1.0/0.6	2.9 (-39, -57, 30)/3.4 (42, -57, 30)
Lingual Gyrus	19	0.1/0.2	2.0 (-9, -85, -3)/3.4 (21, -73, -1)
Superior/Middle Frontal Gyrus	6, 8, 9, 10	1.8/1.0	3.1 (-30, 5, 49)/2.9 (36, 42, 17)
Superior/Inferior Parietal Lobule	7, 39, 40	0.4/0.4	2.6 (-33, -41, 52)/2.4 (33, -41, 57)
Fusiform Gyrus	20	0.1/0.1	2.0 (-39, -16, -24)/2.5 (42, -27, -16)
Insula	13	0.2/0.5	2.4 (-36, 4, 14)/2.3 (39, -2, 17)
Amygdala Gyrus	34	0.3/0.2	2.1 (-28, -5, -13)/2.1 (27, -4, -12)

Table S6. Anatomical information of Asperger's related multimodal brain areas associated with ADOS.

fMRI_IC Area	Brodmann Area	volume (cc)	random effects: Max Value (x, y, z)
Positive			
Superior/Inferior Parietal Lobule	7, 39, 40	2.5/2.1	3.8 (-62, -27, 35)/3.3 (30, -41, 57)
Angular Gyrus	39	0.1/0.3	2.5 (-36, -54, 36)/3.4 (56, -56, 36)
Superior/Middle Temporal Gyrus	21, 22, 38, 42	1.9/1.8	3.3 (-62, -46, 19)/3.2 (62, -18, -2)
Superior/Middle Frontal Gyrus	6, 8, 9, 10	0.3/0.9	2.4 (-18, 43, 42)/2.9 (27, 23, 49)
Negative			
Lingual Gyrus	17, 18, 19	3.3/1.2	3.3 (-9, -67, -2)/3.4 (9, -61, 3)
Fusiform Gyrus	19, 37	0.6/0.2	2.8 (-27, -62, -7)/2.8 (27, -62, -7)
Parahippocampal Gyrus		0.1/0.2	2.0 (-18, -27, -9)/2.4 (33, -38, -6)
Putamen		0.3/0.1	2.4 (-20, -5, 15)/2.3 (15, -2, 19)
**sMRI_IC Area**	**Brodmann Area**	**volume (cc)**	**random effects: Max Value (x, y, z)**
Positive			
Superior/Middle Temporal Gyrus	19, 20, 21, 22, 37, 38, 39, 41	2.4/2.7	5.0 (-42, -49, 16)/3.9 (42, 10, -28)
Superior/Middle Frontal Gyrus	6, 8, 9, 10, 11, 32	1.2/2.0	3.6 (-36, 13, 30)/4.6 (36, 42, 17)
Inferior Parietal Lobule	40	0.5/1.0	3.5 (-50, -36, 38)/3.3 (48, -42, 24)
Insula	13	1.1/1.0	2.8 (-36, -2, 17)/3.5 (33, -25, 21)
Angular Gyrus	39	0.4/0.3	2.9 (-36, -65, 31)/3.1 (45, -57, 30)

Table S7. Anatomical information of PDD-NOS related multimodal brain areas associated with ADOS.

fMRI_IC Area	Brodmann Area	volume (cc)	random effects: Max Value (x, y, z)
Positive			
Fusiform Gyrus	19, 37	0.6/1.0	2.6 (-42, -68, -12)/3.7 (48, -53, -18)
Superior/Inferior Frontal Gyrus	6, 8, 9, 13, 46, 47	0.9/1.1	2.8 (-36, 34, 40)/3.2 (33, 34, 40)
Lingual Gyrus	17, 18, 19	1.7/0.6	3.3 (-27, -85, -6)/3.1 (3, -84, 4)
Angular Gyrus	39	0.4/0.4	2.5 (-42, -65, 31)/3.3 (39, -56, 36)
Superior/Middle Temporal Gyrus	19, 21, 22, 38, 39	0.4/1.3	3.1 (-42, -74, 26)/3.0 (50, -66, 25)
Superior/Inferior Parietal Lobule	7, 39, 40	0.4/0.7	2.8 (-56, -45, 41)/2.8 (42, -59, 42)
Insula	13	0.3/0.5	2.3 (-45, 6, 2)/2.6 (50, -37, 18)
Negative			
Middle Frontal Gyrus	6, 8, 9, 10, 13, 32, 45, 46, 47	1.8/2.2	4.4 (-36, 52, 0)/3.7 (56, 11, 35)
Anterior Cingulate	24, 32	0.3/1.1	2.6 (-15, 47, -2)/3.1 (6, 41, -2)
**sMRI_IC Area**	**Brodmann Area**	**volume (cc)**	**random effects: Max Value (x, y, z)**
Positive			
Superior/Middle Temporal Gyrus	21, 22, 37, 38, 39	1.4/0.6	3.9 (-53, -53, -2)/2.5 (62, -38, -1)
Superior/Middle/Inferior Frontal Gyrus	6, 8, 9, 10, 11, 25, 45, 46, 47	1.4/1.3	3.8 (-36, 36, 23)/3.3 (30, 25, 35)
Fusiform Gyrus	20, 37	0.7/0.1	3.6 (-36, -53, -10)/2.1 (56, -7, -25)
Anterior Cingulate	32	0.4/0.2	3.6 (0, 5, -10)/2.4 (3, 2, -10)
Lingual Gyrus	17, 18	0.3/1.0	2.7 (-21, -73, -4)/3.6 (15, -87, 2)

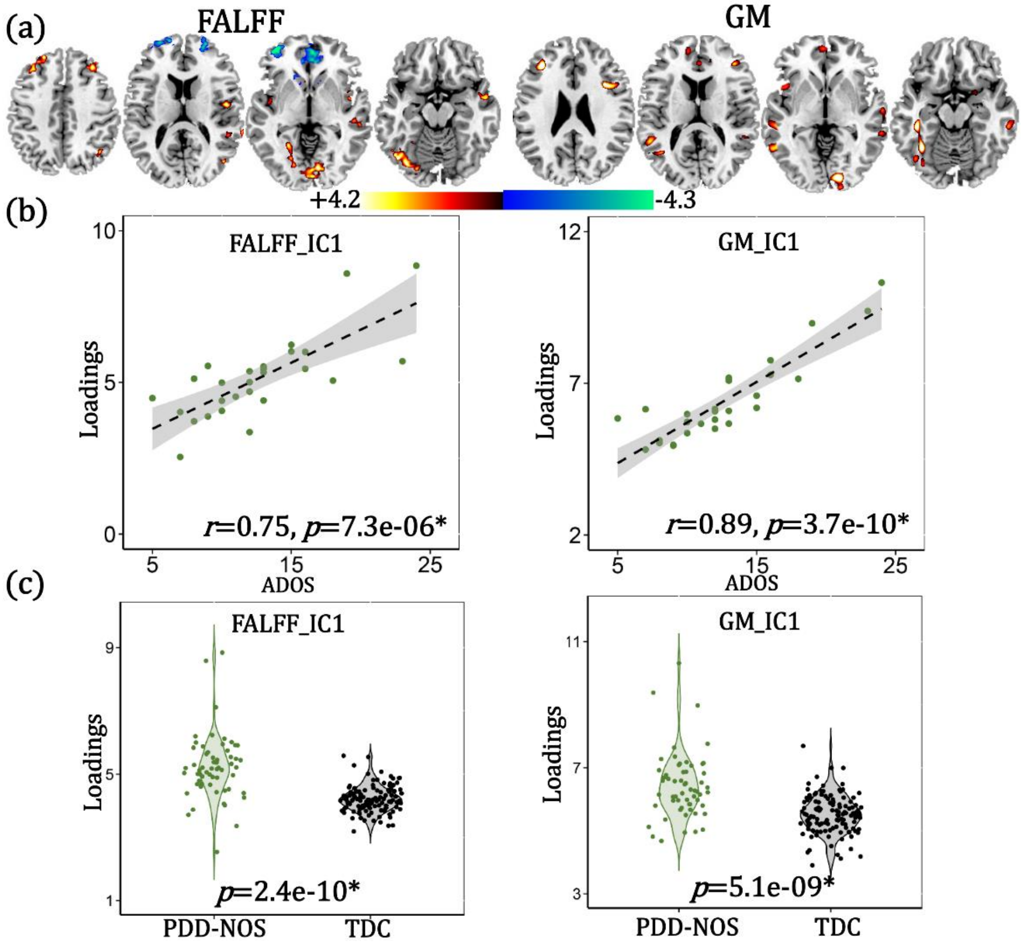

Figure S2. The identified ADOS-associated joint component in PDD-NOS subgroup. (a) The spatial maps are visualized at |Z|>2 thresholds, where the red regions mean positive fALFF or GMV, and the blue areas indicate negative fALFF or GMV. (b) Correlation between loadings of the identified components and ADOS. (c) Group difference between PDD-NOS and controls of the loading parameters.

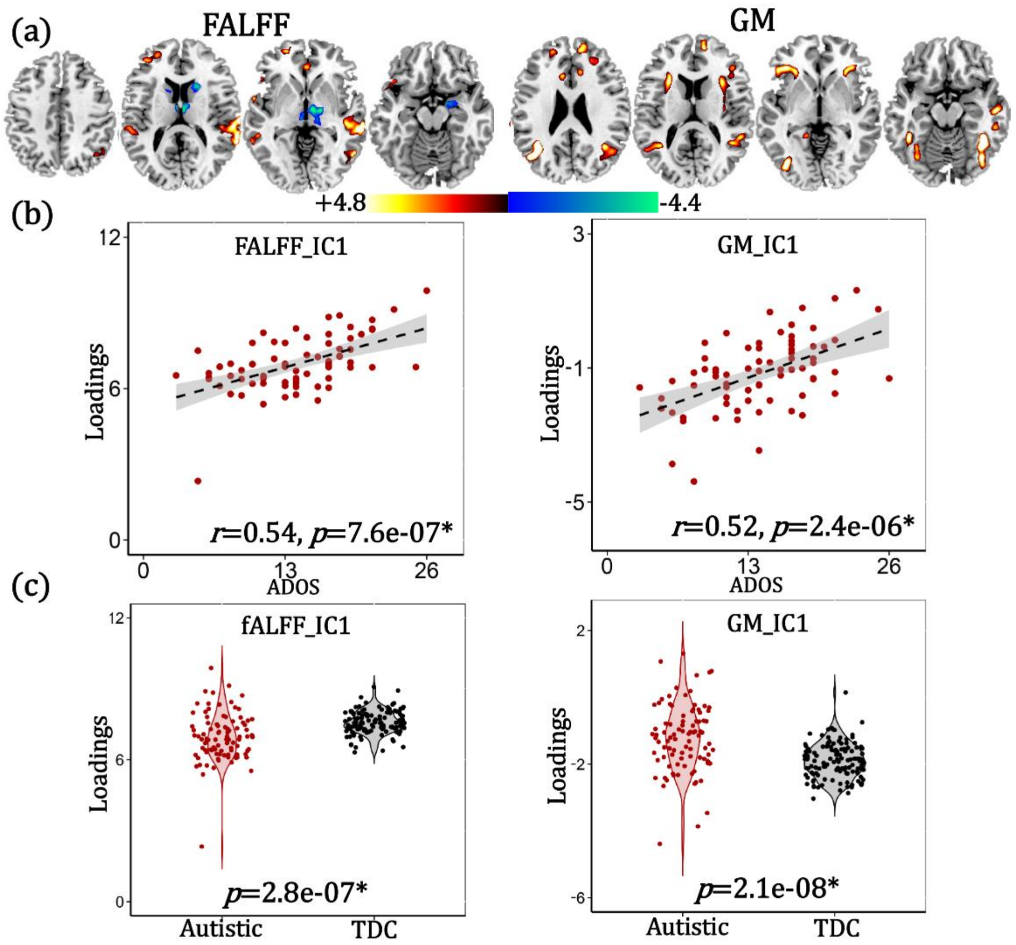

Figure S3. The identified ADOS-associated joint component in Autistic subgroup. (a) The spatial maps are visualized at |Z|>2 thresholds, where the red regions mean positive fALFF or GMV, and the blue areas indicate negative fALFF or GMV. (b) Correlation between loadings of the identified components and total ADOS scores. (b) Group differences between Autistic and controls of the loading parameters (contribution weight of the corresponding component across subjects) of the target component.

Table S8. Anatomical information of Autistic related multimodal brain areas associated with ADOS.

fMRI_IC Area	Brodmann Area	volume (cc)	random effects: Max Value (x, y, z)
Positive			
Superior/Middle Temporal Gyrus	13, 21, 22, 37, 39, 41, 42	0.6/5.1	2.5 (-59, -40, 21)/4.0 (65, -40, 13)
Inferior Parietal Lobule	40	0.5/1.1	3.4 (-45, -45, 41)/3.2 (50, -50, 52)
Superior/Middle/Inferior Frontal Gyrus	9, 10, 11, 46, 47	1.5/0.4	3.1 (-27, 55, 3)/3.2 (18, 60, 30)
Anterior Cingulate	25, 32	0.0/0.3	NaN/2.9 (3, 38, -2)
Lingual Gyrus	18	0.6/0.2	2.7 (-18, -90, 2)/2.4 (30, -79, -6)
Angular Gyrus	39	0.1/0.1	2.0 (-50, -60, 33)/2.6 (48, -65, 36)
Negative			
Caudate		0.3/0.3	2.5 (-15, -22, 20)/2.9 (15, 4, 19)
Thalamus		0.4/0.2	2.4 (-15, -17, 4)/2.7 (9, -12, 1)
Amygdala Gyrus	34	0.1/0.3	2.2 (-15, -12, -15)/2.4 (18, -4, -15)
**sMRI_IC Area**	**Brodmann Area**	**volume (cc)**	**random effects: Max Value (x, y, z)**
Positive			
Superior/Middle/Inferior Frontal Gyrus	6, 8, 9, 10, 13, 44, 45, 46, 47	3.3/3.1	4.5 (-27, 2, 47)/6.3 (30, 14, 38)
Superior/Inferior Parietal Lobule	5, 7, 39, 40	1.5/1.8	5.3 (-42, -30, 37)/4.0 (42, -50, 44)
Superior/Middle Temporal Gyrus	21, 22, 39	1.5/2.1	5.1 (-36, -54, 25)/3.2 (39, -54, 25)
Fusiform Gyrus	20, 37	0.3/0.8	2.6 (-33, -67, -9)/3.9 (42, -53, -12)
Lingual Gyrus	18, 19	1.2/0.6	3.6 (-18, -82, -6)/3.3 (21, -76, -1)
Angular Gyrus	39	0.4/0.5	3.0 (-36, -54, 30)/3.5 (39, -60, 33)
Insula	13, 45	0.6/0.3	2.8 (-30, 18, 10)/2.6 (33, 15, 10)

Table S9. The partial correlations between components' loadings and ADOS after regressing out FD.

	ASD	Asperger's	PDD-NOS	Autistic
fMRI_IC	*r*=0.41, *p*=4.4e-08*	*r*=0.52, *p*=1.1e-05*	*r*=0.75, *p*=1.3e-05*	*r*=0.54, *p*=9.0e-06*
sMRI_IC	*r*=0.35, *p*=4.3e-06*	*r*=0.61, *p*=1.3e-07*	*r*=0.90, *p*=5.0e-10*	*r*=0.52, *p*=3.2e-06*

Table S10. The partial correlations between components and ADOS after regressing out both TR and site.

	ASD	Asperger's	PDD-NOS	Autistic
fMRI_IC	*r*=0.39, *p*=2.7e-07*	*r*=0.59, *p*=0.001	*r*=0.57, *p*=1.1e-06*	*r*=0.55, *p*=5.5e-06*
sMRI_IC	*r*=0.35, *p*=4.5e-05*	*r*=0.83, *p*=2.6e-07*	*r*=0.61, *p*=1.1e-07*	*r*=0.55, *p*=7.6e-07*

Table S11. Group difference with TDC of the identified component after controlling age, handedness, FIQ.

	ASD vs. TDC<br>*p* values	Asperger vs. TDC<br>*p* values	PDD-NOS vs. TDC<br>*p* values	Autistic vs. TDC<br>*p* values
fMRI_IC	2.8e-04	7.4e-26	2.6e-34	1.2e-65
sMRI_IC	3.5e-23	5.5e-07	2.6e-36	3.8e-12

Table S12. Correlation between subtype-related components with age and the mean FD. None of them is significant.

	Age	Mean FD
Asperger's		
fMRI_IC	r=-0.008, p=0.94	r=-0.18, p=0.11
sMRI_IC	r=-0.069, p=0.55	r=-0.057, p=0.62
PDD-NOS		
fMRI_IC	r=-0.12, p=0.39	r=-0.25, p=0.062
sMRI_IC	r=6.0e-05, p=0.99	r=-0.20, p=0.13
Autistic		
fMRI_IC	r=0.008, p=0.94	r=0.061, p=0.56
sMRI_IC	r=-0.036, p=0.73	r=0.072, p=0.50
ASD		
fMRI_IC	r=-0.066, p=0.35	r=-0.08, p=0.23
sMRI_IC	r=-0.042, p=0.52	r=-0.047, p=0.48

Predictive feature extraction

The extracted ROIs (positive and negative brain networks in fMRI_IC, and positive brain networks in sMRI_IC) were used as regressors to predict multiple symptom scores (ADOS and SRS) using an independent sample (ABIDE I) for cross-validation. Specifically, we extracted brain regions from the identified fMRI-sMRI components, which were tested on the possibility to serve as potential biomarkers within each modality. For sMRI, after converting component into Z scores and thresholding at $|Z| >= 2$, positive mask of sMRI_IC was generated. The mask of GM was then used to extract ROI features from every subject. The mean of the voxels within the obtained ROI was calculated for each subject, generating

a $N_{\mathrm{subj}} \times 1$ feature vector from sMRI_IC. For fMRI, positive and negative masks of fMRI_IC were also generated. These masks of fMRI were then used to extract the fALFF features from every subject, generating two $N_{\mathrm{subj}} \times 1$ feature vector for fMRI_IC. Together we form a feature matrix in dimension of $N_{\mathrm{subj}} \times 3$. After biomarker extraction, each of the 3-biomarker vectors and the predicted scores were normalized to mean = 0, std = 1. These vectors were then treated as the linear regressors and the symptom scores were treated as the targeted measures; together, they were put into a multiple linear regression model (equation 2) to obtain a linear equation for an estimate of the target measures. The same procedure was performed for Asperger's and PDD-NOS groups. As for generalized prediction on ABIDE I cohort, the same ROIs identified in ABIDE II were used as masks to extract features from ABIDE I, and the same prediction models trained in ABIDE II were generalized to predict symptom scores of ABIDE I. The predictive accuracy is measured by the correlation between the estimated symptom scores and its true values, as well as the normalized root mean squared prediction error (NRMSE).

Reproducibility across sites

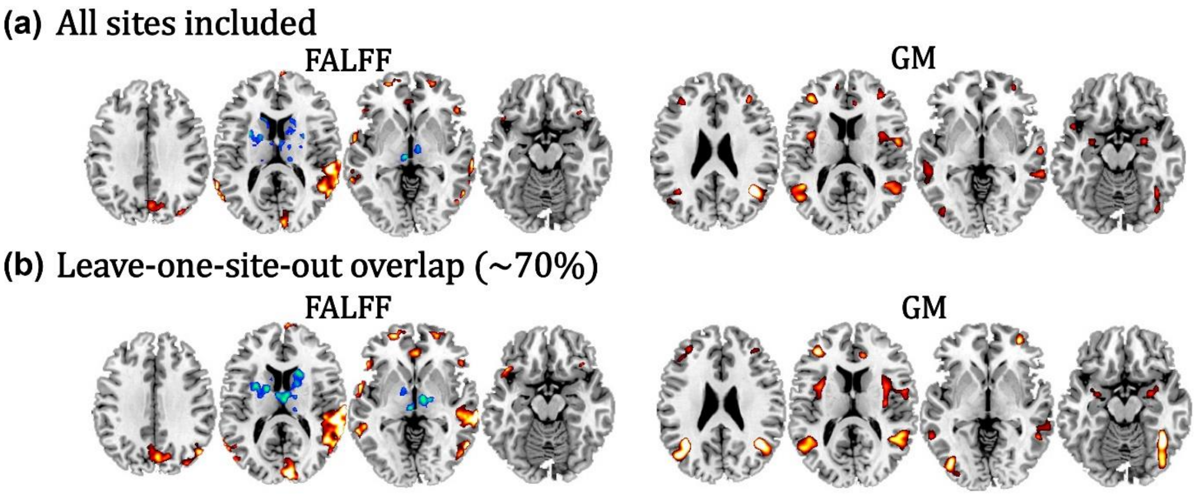

Figure S4. (a) The covarying pattern for the original ASD group. (b) The most frequently occurring (voxels with more than 70% occurrences) covarying pattern by leave-one-site-out.

We used two different ways to discuss replication across sites. The first one is that we performed leave-

one-site-out analysis in the ADOS-guided fALFF and GMV fusion on the whole ASD group to check whether the site effect will contribute to current ASD pattern. As displayed in Table S1, there are 9 sites included in ABIDE II. So, in each analysis of MCCAR+jICA, one site was excluded, and the rest 8 sites were combined in the supervised fusion analysis. This leave-one-site-out procedure was repeated 9 times. Thus, we obtain 9 fALFF+GMV covarying patterns associated with ADOS for the whole ASD group. We also record the number of times each spatial pattern occurs by voxel. Here, we present the most frequently occurring voxels (those which occur more than 70% of sites, i.e., 6 sites among 9) associated with ADOS among these 9 repeated leave-one-site-out analysis, as shown in Fig. S4b. Note that the overlapped model of spatial patterns is highly similar with the ASD pattern (all sites included). This result shows that the altered covarying patterns of fALFF-GMV of DLPFC, superior-middle temporal gyrus and insula in ASD group are consistent across 70% sites in ABIDE II.

The second one is that we performed linear projection of the identified subtype related components from ABIDE II to the corresponding subgroups in ABIDE I to see whether the linkage between subtype related components and ADOS can be replicated in ABIDE I. Take the whole ASD group as an example. ASD related brain maps in ABIDE II were used as spatial maps for ABIDE I, the mixing matrix for ABIDE I can be estimated based on the following linear projection model as in Eq. (7).

$$X_{\mathrm{ABIDE2\_ASD,k}} = A_{\mathrm{ABIDE2\_ASD,k}} \times S_{\mathrm{ABIDE2\_ASD,k}}$$

$$A_{\mathrm{ABIDE1\_ASD,k}} = X_{\mathrm{ABIDE1\_ASD,k}} \times \left(S_{\mathrm{ABIDE2\_ASD,k}}\right)^{-1} \quad k=1,2 \tag{7}$$

where $S_{\mathrm{ABIDE2\_ASD,k}}$ and $A_{\mathrm{ABIDE2\_ASD,k}}$ represent the brain components and the corresponding mixing matrix derived by MCCAR+jICA for ABIDE II ASD group. $X_{\mathrm{ABIDE2\_ASD,k}}$ and $X_{\mathrm{ABIDE1\_ASD,k}}$ represent preprocessed imaging feature matrixes for ASD of ABIDE II and ABIDE I separately, and k represents the modality. The same linear projection was used on Asperger's, PDD-NOS and Autistic subtypes separately. The correlations between components and ADOS in ABIDE I are listed in Table S13. Results show that

the linkage between subtype related components and ADOS detected in ABIDE II can be replicated in ABIDE I age matched cohort.

Table S13. Correlation between ADOS and subtype components by projecting from ABIDE II to ABIDE I cohort.

	ASD	Asperger's	PDD-NOS	Autistic
fMRI_IC	r=0.21, p=0.0031	r=0.38, p=0.01	r=0.76, p=0.03	r=0.21, p=0.0028
sMRI_IC	r=0.35, p=9.1e-04	r=0.35, p=0.025	r=0.85, p=0.007	r=0.24, p=4.6e-04
